# Geometry and physics

**DOI:** 10.1098/rsta.2009.0227

**Published:** 2010-03-13

**Authors:** Michael Atiyah, Robbert Dijkgraaf, Nigel Hitchin

**Affiliations:** 1 School of Mathematics, University of Edinburgh, Edinburgh EH9 3JZ, UK; 2 Institute for Theoretical Physics, University of Amsterdam, Valckenierstraat 65, 1018 Amsterdam, The Netherlands; 3 Mathematical Institute, University of Oxford, 24–29 St Giles, Oxford OX1 3LB, UK

**Keywords:** quantum physics, four-dimensional topology, knot invariants

## Abstract

We review the remarkably fruitful interactions between mathematics and quantum physics in the past decades, pointing out some general trends and highlighting several examples, such as the counting of curves in algebraic geometry, invariants of knots and four-dimensional topology.

## 1. Past history of physics

The relation between mathematics and physics is one with a long tradition going back thousands of years and originating, to a great extent, in the great mystery of the cosmos as seen by shepherds on starry nights. Astronomy in the hands of Galileo ushered in the modern scientific era, and it was Galileo who said that the book of nature is written in the language of mathematics (Drake 1957, p. 237):

Philosophy is written in this grand book, the universe, which stands continually open to our gaze. But the book cannot be understood unless one first learns to comprehend the language and read the characters in which it is written. It is written in the language of mathematics, and its characters are triangles, circles, and other geometric figures without which it is humanly impossible to understand a single word of it; without these one is wandering in a dark labyrinth.

A giant step forwards in using the language of nature to describe physical phenomena was made by Isaac Newton, who developed and applied the calculus to the study of dynamics and whose universal law of gravitation explained everything from the fall of an apple to the orbits of the planets.

The nineteenth century witnessed the greater sophistication of Maxwell’s equations to include the behaviour of electromagnetism, and the twentieth century saw this process take a major step forwards with Einstein’s theory of special relativity and then of general relativity. At this stage both gravitation and electromagnetism were formulated as field theories in four-dimensional space–time, and this fusion of geometry and classical physics provided a strong stimulus to mathematicians in the field of differential geometry.

However, by this time, it had already been realized that atomic physics required an entirely new mathematical framework in the form of quantum mechanics, using radically new concepts, such as the linear superposition of states and the uncertainty principle, that no longer allowed the determination of both the position and momentum of a particle. Here the mathematical links were not with geometry, but with the analysis of linear operators and spectral theory. As experimental physics probed deep into the subatomic region, the quantum theories became increasingly complex and physics appeared to be diverging from classical mathematics, while at the same time dragging analysis along behind on a somewhat rocky journey. Indeed, in the 1950s under a barrage of newly discovered particles, the hope of capturing the fundamental physical laws in terms of deep and elegant mathematics seemed to evaporate rapidly. At the same time there was a strong inward movement in mathematics with a renewed focus on fundamental structures and rigour.

The picture began to change around 1955, ironically the year of Einstein’s death, with the advent of the Yang–Mills equations, which showed that particle physics could be treated by the same kind of geometry as Maxwell’s theory, but with quantum mechanics playing a dominant role. However, it took to the beginning of the 1970s before it became clear that these non-Abelian gauge theories are indeed at the heart of the standard model of particle physics, which describes the known particles and their interactions within the context of quantum field theory. It is a remarkable achievement that all the building blocks of this theory can be formulated in terms of geometrical concepts such as vector bundles, connections, curvatures, covariant derivatives and spinors.

This combination of geometrical field theory with quantum mechanics worked well for the structure of matter but seemed to face a brick wall when confronted with general relativity and gravitation. The search for a coherent framework to combine the physics of the very small (quantum mechanics) with the physics of the very large (general relativity) has, for several decades, been the ‘Holy Grail’ of fundamental physics. The most promising candidate for a solution to this problem is string theory, and the mathematical development of this theory, in its various forms, has been pursued with remarkable vigour and some success.

## 2. The impact of modern physics on mathematics

What has been described so far is the familiar story of the advance of physics necessitating the use and development of increasingly sophisticated mathematics, to the mutual benefit of both fields. Mathematicians have been driven to investigate new areas; and physicists, to quote Eugene Wigner (1960), have been impressed by ‘the unreasonable effectiveness of mathematics in physics’—the remarkable universal properties of mathematical structures.

But over the past 30 years a new type of interaction has taken place, probably unique, in which physicists, exploring their new and still speculative theories, have stumbled across a whole range of mathematical ‘discoveries’. These are derived by physical intuition and heuristic arguments, which are beyond the reach, as yet, of mathematical rigour, but which have withstood the tests of time and alternative methods. There is great intellectual excitement in these mutual exchanges.

The impact of these discoveries on mathematics has been profound and widespread. Areas of mathematics such as topology and algebraic geometry, which lie at the heart of pure mathematics and appear very distant from the physics frontier, have been dramatically affected.

This development has led to many hybrid subjects, such as topological quantum field theory, quantum cohomology or quantum groups, which are now central to current research in both mathematics and physics. The meaning of all this is unclear and one may be tempted to invert Wigner’s comment and marvel at ‘the unreasonable effectiveness of physics in mathematics’.

The key role of physics in many of these areas is to produce an intuitive ‘natural’ context for various abstract mathematical constructions. Pure mathematics not only consists of theorems built step-by-step via logical deductions, but also has an intuitive side—the use of analogy and metaphor to jump from one context to another or to explore new areas to see what *might* be true. Geometrical intuition has often been an important part of this, treating functions as vectors in Hilbert spaces or dealing with the number theory of Fermat’s last theorem with elliptic curves. In the same way classical mechanics has provided a rich setting for the principles of calculus, geometry and more recently dynamical systems. Remarkably, modern physical constructions such as quantum field theory and string theory, which are very far removed from everyday experience, have proven to be a similar fertile setting for mathematical problems. Indeed, in many ways quantum theory has turned out to be an even more effective framework for mathematics than classical physics. Particles and strings, fields and symmetries, they all have a natural role to play in mathematics.

Einstein once said that ‘since the mathematicians have invaded the theory of relativity, I do not understand it myself’ and there is a sense that the reverse invasion has wrong-footed a number of mathematicians. But the presence of physics in contemporary pure mathematics is undeniable and has presented the community with both opportunities and challenges.

This influence manifests itself in two ways. In some sense the easier one is for the mathematician to be presented with a clearly stated conjecture or problem. One then attempts a development of current conventional techniques to provide a rigorous solution. This situation requires a successive distillation of the problem from the physics to pure mathematics, and involves the active participation of committed individuals on the way. It may result in a seriously difficult task, but one that may perhaps be tackled with the mathematician’s toolkit. The construction of instantons in the 1970s, to which we turn in more detail later, was an example of this: a problem derived from quantum physics but refined to one in conventional, but modern, differential geometry. The mathematics that developed from it, and its new viewpoints, was the Fields Medal-winning work of Simon Donaldson.

The second mode of influence is more direct. This is a more fundamental invasion of physical ideas based on the underlying structure and language of quantum field theory—thinking big in terms of path integrals on spaces of fields, for example. The challenge to mathematicians here is much more serious, since it involves developing a sense of intuitive understanding to parallel that of every physicist, for whom this has become second nature since graduate school. It has spurred new ways of thinking in pure mathematics. Clearly, this approach is a long-term programme with many ramifications, somewhat reminiscent of Hilbert’s sixth problem: the axiomatization of all branches of science, in which mathematics plays an important part.

In the following sections we will put flesh on the bare bones of the above remarks by listing a few of the spectacular discoveries in mathematics that have emerged from the interaction with physics.

## 3. Counting curves

Our first example comes from enumerative geometry, a particularly active field in the nineteenth century. In classical geometry many problems concern counting the number of solutions. Starting with the fact that two straight lines in the plane meet in one point (which may be at infinity, in which case the lines are parallel), one goes on to show that curves in the plane given by polynomial equations of degrees *m* and *n* meet in *mn* points, provided we allow complex solutions, points at infinity and count intersections with multiplicity.

A slightly more complicated problem is to count the number of curves in the plane that satisfy certain constraints. One such problem for curves of degree *d*, which are rational, i.e. can be described parametrically by rational functions of one complex variable, is to ask how many such curves go through *n* general points. If *n*=3*d*−1, this number *N*_*d*_ is finite and the problem is to find a formula for *N*_*d*_. If *d*=1 or 2, then *N*=1, but the general formula was not known to classical geometers, and only emerged from physics! Moreover, the methods from physics are powerful enough to deal with more general curves, and also with more general problems of the same type (Kontsevich & Yu 1994; Ruan & Tian 1995).

An even richer example is the counting of rational curves not on the plane, but on a quintic hypersurface *X* given by the equation

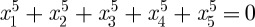

in projective four-space. This equation can be seen to describe a manifold of three complex dimensions, or six real dimensions. The quintic *X* plays an important role in string theory. It is an example of a Calabi–Yau space, a special class of complex manifolds that allow for a solution of the Einstein equations of gravity in empty space. In string theory *X* can be used to compactify the 10-dimensional space–time down to the four dimensions of physics.

Computing the number *N*_*d*_ of degree-*d* rational curves on *X* is a notoriously difficult problem, since its complexity grows exponentially as a function of the degree *d*. For example, the number *N*_1_=2875 of lines on the quintic is a classical result dating from the nineteenth century. The next number to be computed, *N*_2_=609 250, counts the different conics in the quintic and was only found around 1980. Finally, the number of twisted cubics, *N*_3_=317 206 375, was the result of a complicated computer program. So one can easily imagine the considerable impact in the mathematics community when physicists announced that they were able to compute all these numbers (Candelas *et al.* 1991). For example, the first 10 such numbers are listed in table 1.

**Table 1.** The first 10 numbers *N*_*d*_.

*d*	*N*_*d*_
1										2875
2									609 250
3								317 206 375
4							242 467 530 000
5						229 305 999 987 625
6					248 249 742 118 022 000
7				295 091 050 570 845 659 250
8			375 632 160 937 476 603 550 000
9		503 840 510 416 985 243 645 106 250
10	704 288 164 978 454 686 113 488 249 750

How were physicists able to solve this difficult problem without actually doing the computations in algebraic geometry? The short answer is: by placing the enumerative problem in the right physical context. Within quantum theory it makes perfect sense to combine all the numbers *N*_*d*_ into a single generating function

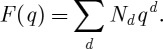

In fact, this function has a straightforward physical interpretation. It can be seen as a probability amplitude for a string to propagate in the Calabi–Yau space *X*. In quantum theory one has to operate under the fundamental principle of summing over all possible histories with a weight given by the classical action. In that spirit a quantum string can be thought to probe all possible rational curves of every possible degree *d* at the same time, with weight *q*^*d*^. The classical limit is recovered when we send the parameter *q* to zero, in which case only the degree *d*=0 curves contribute, i.e. the points of *X*.

But there was a second ingredient that helped to find the physical solution. There exists an equivalent formulation of this physical process using a so-called mirror Calabi–Yau manifold *Y* . As far as classical geometry is concerned, the spaces *X* and *Y* are very different; they do not even have the same topology. But in the realm of quantum theory, they share many properties. In particular, with a suitable identification, the string propagation in spaces *X* and *Y* are identical. The interchange of *X* with *Y* is called mirror symmetry (Cox & Katz 1999; Hori *et al.* 2003). It is a typical example of a quantum symmetry. Moreover, the difficult computation of the function *F* on the manifold *X* (known by physicists as the A-model) turned out to translate into a much simpler expression on the mirror manifold *Y* , where it could be captured by a so-called period integral (known as the B-model). These integrals are a well-known element in the theory of variation of complex structures in algebraic geometry. It is mirror symmetry that easily leads to the above table of numbers.

Mirror symmetry is an example of a much broader area of mathematics influenced by physics: symplectic geometry—the branch of differential geometry based on the underlying structure of Hamiltonian mechanics. Gromov’s theory of pseudoholomorphic curves in symplectic manifolds had already shown that, at the internal low-dimensional level, algebraic and symplectic geometry had common features, but the string theorists’ notion of mirror symmetry brought the parallels between symplectic and algebraic geometry into much sharper focus. The A-model and the B-model for Calabi–Yau manifolds were simply two sides of a common quantum theory. Classically, the A-model was symplectic geometry and the B-model was algebraic geometry, but mirror symmetry gave the same structure to their moduli spaces, if one included the behaviour of Gromov’s curves.

In Kontsevich’s formulation of this phenomenon—homological mirror symmetry (Kontsevich 1995)—the natural subspaces on either side of the algebraic/symplectic divide (algebraic or Lagrangian subvarieties and bundles over them) are supposed to generate equivalent mathematical objects. Finding the framework for proving this mathematically is currently a topic of great interest.

Mirror symmetry is a good example of a more fundamental influence of physics on geometry, one which involves a significant change of viewpoint on the part of the pure mathematician. The functional integral and the partition function are the bread and butter of the theoretical physicist, but they go counter to the traditional mathematical approach to a problem. Take the algebraic geometer studying algebraic curves in an algebraic variety, like the quintic threefold we have just seen. Traditionally, the mathematician restricts to a fixed topological invariant of the curve, for example its degree *d*. The physicist, by contrast, studies all curves at once—the degree being some indexing of terms in a series expansion of the partition function—and by doing so sees relations that are invisible in a step-by-step analysis. The formulae above count rational curves, curves of genus zero, but one may also use the topological invariant of the genus as a parameter in a generating function, and there the physicist sees further relations. The very idea (as occurs in Gromov–Witten theory) that there is a link between counting higher-genus curves and those of genus zero is a very radical one for the mathematician to comprehend.

Supersymmetry, the link between bosons and fermions, is a closely related concept from physics that has also influenced differential geometry. As first noted by Edward Witten, supersymmetry applied within quantum mechanics is an elegant way to derive the basic principles of Morse theory (Witten 1982). Another application is in the development of hyper-Kähler geometry—the curved manifestation of Hamilton’s quaternions. Although the definition has been in the differential-geometric literature since the 1950s, it was 30 years later, as a result of the infiltration of ideas from the supersymmetric sigma model, that a mechanism for constructing good examples was found. There are many spaces under current investigation that have a dual interpretation—one in terms of the differential geometry of moduli spaces and the other derived from supersymmetric field theories. These have now penetrated pure mathematics in algebraic and number-theoretic areas such as the geometric Langlands programme and representation theory. Supersymmetry has also generated further differential-geometric structures, sometimes long before the mathematicians were ready to listen. One example, that of a generalized complex structure, is a conventional structure that unifies both symplectic and complex geometry and retains in classical form some of the consequences of supersymmetry.

## 4. Knot invariants

For our second class of examples we move to three-dimensional topology. The prime example of a topological problem, where length is irrelevant because we allow deformation or stretching (but not cutting), is that of knots in three-dimensional space. These are familiar to all of us and we easily recognize their complexity. For example, if we consider two closed knots (with no loose ends) it is not easy to tell whether we can deform one to look like the other. A useful tool to distinguish knots is provided by a numerical invariant: the knot invariant. This is a number that can be computed by a formula from the picture of a knot, as a piece of string laid on a table, but which is unchanged if we turn the knot around and place it back in a different pattern.

One such invariant, known since the 1920s and for a long time the only one, is the Alexander polynomial. This expression in one variable *t* is of the form

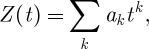

where *k* runs over a finite set of integers (positive or negative) and the coefficients *a*_*k*_ are integers. These *a*_*k*_ actually provide a string of invariants, but it is sensible to turn them into a polynomial *Z*(*t*).

In 1984 the world of knot theory underwent a remarkable new development when Vaughan Jones discovered a polynomial knot invariant (now named after him) that was different from the Alexander polynomial (Jones 1985). Crucially, it was chiral, that is, it could distinguish knots from their mirror images, which the Alexander polynomial could not. It soon emerged that this new invariant was part of a grand family of invariants based on Lie algebras and their representations.

The Jones polynomial emerged from a mysterious background of mathematics and physics and seemed to fit into no general framework. But shortly afterwards, Witten showed how to interpret the Jones polynomial in terms of a quantum field theory in three dimensions (Witten 1989).

In fact, this relation between knot invariants and particles goes to the very beginning of relativistic quantum field theory as developed by Feynman and others in the 1940s. The basic idea is that, if we think of a classical particle moving in space–time, it will move in the direction of increasing time. However, within quantum theory the rules are more flexible. Now a particle is allowed to travel back in time. Such a particle going backwards in time can be interpreted as an anti-particle moving forwards in time. Once it is allowed to turn around, the trajectory of a particle can form, as it were, a complicated knot in space–time. The rules of quantum theory will associate to each such trajectory a probability amplitude that describes the likelihood of this process actually taking place.

Feynman was the first to realize that the processes of particle physics can be captured by these diagrams. For an interacting quantum field theory, the diagrams will actually form complicated ‘coloured’ graphs. If one produces an actual closed loop, as in a mathematical knot, such a diagram is called a vacuum amplitude. The particle travelling along the loop cannot be detected experimentally—the corresponding Feynman diagram describes a so-called virtual particle. Yet, these diagrams describe a clear physical effect: the quantum contributions to the vacuum energy. In order to establish contact between this formulation of particle physics and knot theory, Witten had to replace the usual four dimensions of space–time with three dimensions and work with a special type of gauge theory based on the Chern–Simons topological invariant. The full theory allowed for many choices, such as the gauge group, the representation of the particles associated to the knot and coupling constants.

Relating quantum field theory to knot invariants along these lines had many advantages. First, it was fitted into a general framework familiar to physicists. Second, it was not restricted to knots in three-dimensional Euclidean space and could be defined on general three-dimensional manifolds. One could even dispense with the knot and get an invariant of a closed three-dimensional manifold. Such topological invariants are now called quantum invariants and have been extensively studied. The chirality of these invariants is then closely related to the chirality of Yang and Lee in weak-interaction physics.

This example of quantum knot invariants makes clear that physics bridges in a natural way the realms of geometry and algebra. Indeed, quantization can be seen as taking a geometric object, say a curve in a Calabi–Yau space or a knot in a three-manifold, and associating to it an algebraic object, in these examples the complex number that represents the probability amplitude of the corresponding quantum process. Within the algebraic context, new tools such as recursion relations and generating functions become available and can shed new light on geometry.

## 5. Donaldson invariants

For our third example we move to four dimensions. In 1983 Simon Donaldson applied ideas from physics to make a spectacular breakthrough in our understanding of four-dimensional geometry. He showed that there were some very subtle invariants in four dimensions, not present in any other dimension, which were preserved under smooth deformation but not under general continuous deformation (Donaldson & Kronheimer 1990). Indeed, dimension four is special not only for physics, where it represents space–time, but also for geometry, where there are unique phenomena associated to this particular dimension.

Donaldson found his invariants by studying special solutions of the Yang–Mills equations, which had already been introduced by physicists under the name of ‘instantons’. These solutions have the property that they are essentially localized to a small region in space–time, thereby describing an approximately instantaneous process. Instantons had a family resemblance to the ‘solitons’ or solitary waves first observed by John Scott Russell in the early nineteenth century.

Donaldson’s work originated in physics in the same way as the Hodge theory of harmonic forms had been inspired by Maxwell’s equations, but this connection appeared rather formal. More importantly, within Yang–Mills theory, the equations become nonlinear. Instead of studying the solutions themselves, Donaldson put in a central position the so-called moduli space that parametrizes the family of solutions. In fact, one could regard the gauge-theoretic approach to four-dimensional differential topology as replacing the points of the four-manifold by nonlinear instanton solutions of the Yang–Mills equations.

Moduli spaces are an essential ingredient of modern mathematics dating back to the early nineteenth century. Those days were dominated by the theory of elliptic functions. These are analytical functions of a simple complex variable that have two independent periods. The ratio is called the modulus and is the unique parameter of the theory. Again in the nineteenth century attention shifted to the multivariable generalization with a lattice of periods that provide many moduli and lead to the famous theta-functions. In the twentieth century it was realized that there is a nonlinear generalization of this theory, but the nonlinearity leads to complicated moduli spaces about which little was explicitly known. Even their topological properties were not known.

The development of non-Abelian gauge theories, such as Yang–Mills theory, both by differential geometers and by physicists, has led to substantial progress in our understanding. We now know much more about these nonlinear moduli spaces and much of the insight and progress has come from quantum field theory and string theory.

Donaldson’s work involved a melting pot of ideas, not so much from physics, but from the mathematical areas of nonlinear analysis of partial differential equations, differential and algebraic geometry, and topology. Nevertheless, the whole idea of studying a moduli space in this context—a space of connections up to gauge equivalence—had an essential physics origin. The characters were familiar but less so the role that they had to play.

Again it was Witten who showed that Donaldson’s invariants could be interpreted in terms of a quantum field theory and that this would have profound consequences. Moreover, this field theory was a close cousin of the standard theories used by particle physicists, except that it had a ‘twist’ that produced topological invariants, not dependent on the intricate details of the underlying geometry of space–time.

This interpretation of Witten paid handsome dividends a few years later when Seiberg and Witten showed that the corresponding physical theory could be solved in terms of a much simpler structure. Yang–Mills theory is fundamentally based on a choice of non-Abelian Lie group, usually taken to be the group *SU*(2). The non-commutativity of this symmetry group leads to the nonlinearities of the associated partial differential equations. However, in physics it was known that in quantum theories these non-Abelian symmetries often manifest themselves only at very short distance scales. At large distances the symmetry can be broken to a much simpler Abelian group. For example, in the case of *SU*(2) only the circle group *U*(1) of electromagnetism would appear together with possibly some charged matter particles.

Seiberg and Witten were able to make this physical intuition precise for the class of twisted supersymmetric quantum field theories relevant for the Donaldson invariants (Seiberg & Witten 1994). The resulting Seiberg–Witten invariants were based on a *U*(1) gauge field interacting nonlinearly with a spinor field (Witten 1994). These invariants again involved characters familiar to the mathematician, Dirac operators and Spin^*c*^ structures, and this area was the focus of intense research activity in the 1990s. It seemed as if results that were difficult to prove using Donaldson theory were easier here and vice versa. The pay-off in mathematics for the appeal to the physicist’s intuition was clear: one had a new tool for studying four-dimensional manifolds. On the other hand, to establish in conventional terms that really there was a link between the two theories proved to be an enormous task, only recently accomplished.

## 6. Dualities

More recent developments have revolved around topological string theory on Calabi–Yau threefolds, relating them to invariants of sheaves and bundles. In string theory one considers not only rational curves, but Riemann surfaces of general topology. This leads to generating functions *F*_*g*_, where *g* is the number of holes or genus of the surface, that generalize the function considered in §3. Out of these one can make a master generating function,

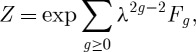

that can be seen to count contributions of all possible topologies, including disconnected surfaces. The parameter *λ* is called the string coupling constant. These string theory invariants are a rich area for mathematical connections. In particular, for toric Calabi–Yau spaces they can be computed exactly.

There is remarkable re-interpretation of these string partition functions linking them directly to the so-called Donaldson–Thomas invariants of sheaves on the underlying Calabi–Yau space. Roughly, one re-expresses the function *Z* in terms of a Fourier series

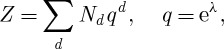

where the coefficients *N*_*d*_ now are assumed to equal the Donaldson–Thomas invariant of certain sheaves.

As an illustration we can consider the simplest possible Calabi–Yau manifold: complex three-space **C**^3^. In this case the quantity *F*_*g*_ can be computed (it is a rather complicated intersection number on the moduli space of Riemann surfaces) as

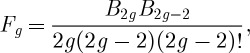

where the *B*_*n*_ are the Bernoulli numbers defined by

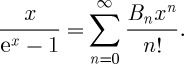

Plugging these numbers into the expression for *Z* gives a remarkable result

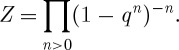

This expression has a direct combinatorial interpretation in terms of a weighted sum over the so-called plane partitions that can be related to ideal sheaves on **C**^3^.

This example illustrates a much more general property of quantum invariants. Often they are captured by a generating function that allows two different expansions. These two expansions and the corresponding interpretations are an example of a duality transformation. Such dualities point to deep symmetries underlying quantum field theory. Naively, a quantum field theory is described by a path integral over a space of classical fields. However, many phenomena such as duality symmetries are not readily explained within that framework. In fact, it is well known in physics that a single theory can have various weakly coupled descriptions in terms of very different sets of fields. Even fundamental properties such as the gauge group are not invariant.

Other ‘quantum results’ involve seeing structure in algebraic topology by packaging cohomology groups of related spaces together. There are many examples of this type of activity. A beautiful case is the S-duality of a special class of 𝒩=4 supersymmetric Yang–Mills theories (Vafa & Witten 1994). These encode a set of invariants that are close cousins of the Donaldson invariants. The generating function of these theories takes the form

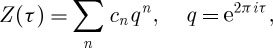

where *n* keeps track of the instanton number or second Chern class. The invariant *c*_*n*_ is then computed in terms of some subtle intersection theory on the moduli space of bundles with this characteristic class. The function *Z* has a remarkable symmetry property. It is invariant under the transformation *τ*→−1/*τ*. More generally, it turns out to be given by a modular form for the group *SL*(2,**Z**). These symmetries obviously impose strong constraints on the coefficients *c*_*n*_.

Directly related to this are the S-duality conjectures of Sen (1994) on what are to the mathematician spaces of rational functions *p*/*q*, where *q*=*z*^*k*^+*a*_2_*z*^*k*−2^+…+*a*_*k*_ and the resultant of *p* and *q* is one. The action of a *k*th root of unity *ω* picks out eigenspaces in the cohomology with eigenvalue *ω*^ℓ^ for 0≤ℓ≤*k*. The S-duality property implies that on the sum of these cohomology spaces for all *k* there is an action of *SL*(2,**Z**) compatible with linear transformations of the vector (*k*,ℓ). This linkage between parts of the cohomology of *different* spaces is quite alien to the traditional topologist, but can in fact be shown mathematically.

The group *SL*(2,**Z**) and modular forms appear frequently also in what are called ‘wall-crossing’ formulae for Donaldson invariants—jumps in the invariants when passing from one generic regime to another. Wall-crossing in other moduli problems has generated a new area in geometry where the naive description of a partition function as a generating function for counting objects has to be replaced by more sophisticated ways of counting and encapsulating the data.

## 7. Thinking big

One problem for mathematicians is that the functional integrals that form the basis of many of the above approaches, and in which the many exotic types of symmetries are more obviously present, are as yet not rigorously defined.

The rigorous study of quantum field theories is a very hard problem and has been slow in development, even for theories much simpler than those that impact on geometry. Even though quantum field theory is used in physics every day, the mathematical foundations underlying the standard model of particle physics still have to be constructed. Even the rigorous treatment of pure quantum Yang–Mills theory without matter is still open and, in fact, is one of the seven Millennium Prize Problems of the Clay Mathematics Institute (Carlson *et al.* 2006).

So, instead of reverting to the origins of these new ideas, mathematicians are now developing their own axiomatic versions, in particular of topological quantum field theories, to suit their own ends. This is not unusual in mathematics—instead of asking what the real numbers really *are*, we are happier to characterize them by their properties, which we can use on an everyday basis.

This mathematical approach has uncovered a rich structure. For example, a topological quantum field theory requires a hierarchy of concepts, the lower levels of which are quite familiar, but progress requires some tough mental activity in getting a feeling for new objects. In an *n*-dimensional theory, one associates to an *n*-dimensional manifold *M* without boundary a number, and to an (*n*−1)-dimensional manifold *N* without boundary a vector space *H*. If a manifold has a boundary *N* then it defines a vector in *H*. When a manifold *M* is cut in two along *N*, the inner product in *H* of the two vectors from the two halves is the invariant. These are the lower, comprehensible, levels.

The problem arises when *N* itself has a boundary (so that *M* is constructed from objects with ‘corners’) and then the theory becomes more complicated and requires, or so it seems, the concepts of higher-order category theory. A generation of mathematicians is now being raised on these ideas. It is no coincidence that the algebraic topologists, for whom cohomology theories, axioms and huge spaces such as spectra are familiar objects, are the ones who are using them most. The categorical language needed, and fed back to the physicists, often involves that of mathematicians such as Grothendieck, whose highly abstract and general work of the 1950s reformulated algebraic geometry.

Having said that, the best-known topological quantum field theory—the Chern–Simons theory, which explains in quantum terms the Jones polynomials of knots—can so far be constructed rigorously only in a very down-to-earth way in terms of generators and relations, rules that tell you how to calculate. Alternatively, there is a combinatorial formula using a statistical ensemble, where one essentially makes a finite model of the underlying geometry. We have here the unsatisfactory situation where the physicist proposes a functional integral—an average over all fields—which, if well defined, clearly describes a topological invariant. On the other hand, to establish that it defines a topological quantum field theory satisfying the axioms, we have to follow the implications of taking a knot, projecting it on a plane and stating what happens to the Jones polynomial under some basic moves. The nitty-gritty of combinatorics is the only rigorous hold we have on a general theory.

## 8. The future?

One of the issues that divides mathematicians and physicists is the degree of evidence needed to support a claim. The fact that 10^13^ zeros of the Riemann zeta function lie on the critical line has little impact on a mathematician, while that many experimental verifications might seem substantial evidence to a physicist. So a number of calculations (with quite surprising results for the mathematician) were carried out by physicists on specific varieties to test mirror symmetry, but the degree of generality is still not clear. There are nevertheless some rigorous theorems exhibiting the presence of mirror symmetry for large classes of manifolds, especially in the so-called toric case.

In fact, there does seem to operate some sort of complementarity (in the spirit of Niels Bohr) that makes it difficult to combine physical intuition with mathematical rigour. The mathematical proofs of conjectures often proceed not by making the physical intuition more precise, but by taking completely alternative routes, familiar to mathematicians but foreign to physicists.

Recent ideas from quantum gravity and string theory challenge the fundamental concepts of geometry at an even deeper level. Physical intuition tells us that the traditional pseudo-Riemannian geometry of space–time cannot be a definite description of physical reality. Quantum corrections in the theory of gravity will change this picture at distances of the order of the Planck scale. Familiar fundamental properties such as locality only appear at much larger scales. In fact, there is now much evidence within string theory—usually referred to as ‘holography’—that in the end geometry itself is an emergent quantity. The classical laws of gravity only appear in the limit where the number of degrees of freedom of the underlying quantum theory is taken to infinity, very similar to the emergence of the macroscopic laws of thermodynamics out of the microscopic description of statistical mechanics. The definite mathematical formulation of such a concept of ‘quantum geometry’ is however still far away.

One could also question whether there exists a single overarching mathematical structure that captures all these aspects of quantum theory, or whether one is simply dealing with a combination of different complementary points of view, like the charts and maps of a manifold. As a whole, the study of quantum geometry takes on the form of a rich mathematical programme, very much like the Langlands programme, with many non-trivial examples, strange relations, dualities and automorphic forms, tying together diverse fields, with vast generalizations, all in an open-ended project that seems to encompass more and more mathematics.

Whatever the successes and failures of string theory or supersymmetry in the experimental domain, it is clear that the impact on mathematics, and on geometry in particular, is permanent. Yet, there is no evidence of the existence of a hybrid between the theoretical physicist and the mathematician. It may be that string theorists can be accused of producing theories without any practical evidence, but what they feed to mathematics is firmly rooted in a particular view of the universe and what drives it, a view that is not yet part of a mathematician’s development. Recent history shows how these distinct viewpoints work together for our mutual benefit, producing some of the most exciting and surprising results in mathematics. Given the rich grounds still to be explored, it seems likely that they will continue to do so for some time yet.
